# Derivatives of L-Ascorbic Acid in Emulgel: Development and Comprehensive Evaluation of the Topical Delivery System

**DOI:** 10.3390/pharmaceutics15030813

**Published:** 2023-03-02

**Authors:** Aleksandra Stolić Jovanović, Milica Martinović, Ana Žugić, Ivana Nešić, Tomislav Tosti, Stevan Blagojević, Vanja M. Tadić

**Affiliations:** 1 “Filly Farm” Pharmacy, Miloša Velikog bb, 11320 Velika Plana, Serbia; 2 Department of Pharmacy, Faculty of Medicine, University of Nis, Boulevard Dr. Zorana Djindjića 81, 18000 Nis, Serbia; 3 Department for Pharmaceutical Research and Development, Institute for Medicinal Plant Research “Dr. Josif Pančić”, Tadeuša Koscuška 1, 11000 Belgrade, Serbia; 4 Faculty of Chemistry, University of Belgrade, Studentski trg 12-16, 11158 Belgrade, Serbia; 5 The Institute of General and Physical Chemistry, Studentski trg 12/V, 11158 Beograd, Serbia

**Keywords:** L-ascorbic acid, ascorbyl palmitate, magnesium ascorbyl phosphate, emulgel, Franz diffusion cell, in vivo study, texture and sensory analysis

## Abstract

The dual controlled release of emulgels makes them efficient drug delivery systems of increasing interest. The framework of this study was to incorporate selected L-ascorbic acid derivatives into emulgels. From the formulated emulgels, the release profiles of actives were evaluated considering their different polarities and concentrations, and consequently their effectiveness on the skin via a long-term in vivo study that lasted for 30 days was determined. Skin effects were assessed by measuring the electrical capacitance of the *stratum corneum* (EC), trans-epidermal water loss (TEWL), melanin index (MI) and skin pH. In addition, the sensory and textural properties of emulgel formulations were compared with each other. The changes in the rate of the release of the L-ascorbic acid derivatives were monitored using the Franz diffusion cells. The obtained data were statistically significant, and indicated an increase in the degree of hydration of the skin and skin whitening potential, while no significant changes in TEWL and pH values were detected. The consistency, firmness and stickiness of the emulgels were estimated by volunteers applying the established sensory evaluation protocol. In addition, it was revealed that the difference in hydrophilic/lipophilic properties of L-ascorbic acid derivatives influenced their release profiles without changing their textural characteristics. Therefore, this study highlighted emulgels as L-ascorbic acid suitable carrier systems and one of the promising candidates as novel drug delivery systems.

## 1. Introduction

Designing a new promising skin drug delivery system is always challenging task. It is crucial to attain the appropriate stability, applicability, and the rate of release of the active ingredient. The topical drug delivery systems vary in formulation and range in consistency from liquid to powder, but the most popular products are semi-solid preparations. Currently, emulgels are affordable and effective as new delivery options with enhanced stability [1].

The presence of a gelling agent in the water phase converts a classical emulsion into an emulgel, thus creating a new class of dosage forms with many advantages. In this way, the typical problems associated with emulsions, such as phase separation and creaming, are resolved, and stability is improved. Their use has been expanded both in cosmetics and in pharmaceutical preparations due to the favorable properties they possess: emulgels are greaseless, thixotropic, easily spreadable, emollient, easily removable, non-staining, water-soluble, transparent, bio-friendly, and have a longer shelf life and attractive appearance [2,3]. In addition, some of their advantages are better loading capacity, selectivity to a specific site, and suitability for medications with a short biological half-life and a narrow therapeutic window. Emulgels have emerged as novel vehicles for drug delivery that improve patient compliance and have shown suitability in the case of self-medication, with the ability to discontinue their use without consequences when required [4,5,6,7,8]. Furthermore, emulgels provide a solution for loading hydrophobic drugs in a water-soluble gel base [2]. Hence, the therapeutic hydrophobic moieties can be incorporated into the oily phase and delivered easily through the skin [4,9]. The aforementioned properties of emulgels make them more efficient and productive compared to other topical delivery systems. Thus, the delivery of a greater number of topical drugs in the form of emulgels seems to be possible in the future [10].

Due to its diverse spectrum of beneficial properties on the skin, L-ascorbic acid is a molecule widely used in numerous cosmetic and dermatological products. Specifically, it is an anti-aging molecule [11] with potent antioxidant activity, with the ability to neutralize oxidative stress caused by different factors (exposure to UV radiation, etc.) [12]. Its antioxidant properties are responsible for clinical applications, ranging from photoprotection to antipigmentation. L-ascorbic acid is a vital component of collagen biosynthesis, as it acts as the enzymatic cofactor for prolyl hydroxylases, which are essential enzymes for crosslinking and stabilizing the collagen fibers. In a variety of inflammatory dermatoses, the use of L-ascorbic acid is beneficial in enhancing the cohesion in the dermal-epidermal junction [12,13]. In studies to date, this molecule has been shown to be successfully applied in the treatment of hyperpigmentation and melasma. By affecting the active site of tyrosinase, an enzyme necessary for melanin production, it acts as a skin-lightening agent [14]. It was demonstrated that topically applied L-ascorbic acid reduces post laser-resurfacing erythema and decreases acne appearance and acne scarring [13]. Due to its high susceptibility to environmental conditions, the greatest obstacle in utilizing this molecule for incorporation in pharmaceutics, cosmetic products and food is preserving its stability. The instability of L-ascorbic acid is especially noticeable in aqueous solutions, where degradation to furfural is the main degradation pathway. Recent studies suggested that the degradation of L-ascorbic acid is concentration-dependent, i.e., more pronounced in lower concentrations [15,16,17]. The traditional approaches (creams, emulsion and lotions) limit the great effectiveness of L-ascorbic acid in skin care and contribute to its relatively poor stability [11]. Its limitations with regard to practical applications are being overcome by more stable derivatives with different chemical properties on the one hand, and by the development of suitable carrier systems on the other [18]. Recent studies revealed that L-ascorbic acid-loaded self-double-emulsifying drug delivery system (SDEDDS)–based hydrogels displayed a promising approach for its topical application. An in vitro permeation investigation showed that this type of drug delivery system could significantly enhance permeation through and into the skin. Furthermore, the conducted in vitro release study showed a sustained release pattern of L-ascorbic acid from the examined hydrogels [19]. Zhou et al., investigated pectin-coated L-ascorbic acid liposomes as a transdermal drug delivery system with the advantages of better storage stability and skin permeation [20]. Kim and coworkers demonstrated that the addition of glycerine and propylene glycol is useful in preventing oxidation and preserving L-ascorbic acid in cosmetic emulsions [21]. In the cosmetic and food industry, esters of L-ascorbic acid are its preferable form, especially when encapsulated into microemulsions, polymeric nanoparticles, bilayer vesicles, and solid lipid nanoparticles [22,23,24]. Ascorbyl palmitate and magnesium ascorbyl phosphate are widely used L-ascorbic acid derivatives with different hydrophilic/lipophilic properties that affect their ability to penetrate the skin [18]. The efficacy of these derivatives is concentration-dependent, with the type of carrier system playing a particular role in their dermal application [25].

Considering the abovementioned facts, this study aimed to develop emulgels as potential delivery systems for L-ascorbic acid derivatives of different polarities and concentrations that possess the appropriate stability, sensory properties, and skin effects. Therefore, two series of emulgels have been formulated in two different concentrations, one containing ascorbyl palmitate, and the other containing magnesium ascorbyl phosphate, as the active ingredients. Afterward, a mutual comparison in an in vitro study with respect to their stability, textural characteristics, and release profiles was conducted, and was further complemented by the in vivo evaluation of their skin effects and sensory properties.

## 2. Materials and Methods

### 2.1. Materials

For the preparation of the investigated emulgels, the L-ascorbic acid derivatives ascorbyl palmitate and magnesium ascorbyl phosphate were used as active ingredients (AvenaLab Cosmetics, Vršac, Serbia). The following reagents were used as oil phase components: Myritol^®^ 318 from Henkel (Düsseldorf, Germany), MontanovTM82 and MontanovTM14 from Seppic (La Garenne-Colombes, France), Paryol 165 OL/R from Comcen (Belgrade, Serbia), and isopropyl myristate from Centrohem (Stara Pazova, Serbia). The aqueous phase consisted of propylene glycol (Fagron, Rotterdam, Netherlands) and hydroxyethylcellulose (AvenaLab Cosmetics, Vršac, Serbia). As a preservative, Euxyl PE 9010 (phenoxyethanol (and) ethylhexylglycerin (90:10), from Comcen, Serbia) were employed. Purified water was obtained from the Faculty of Medicine (University of Niš, Serbia), while a BlueClearRO600P reverse osmosis water cleaner system with integrated BlueSoft07-MB mixed bed salt remover (Euro-Clear Ltd., Gönyű, Hungary) was used for obtaining the ultra-pure water. Phosphoric acid solution (49–51%), L-ascorbic acid, sodium phosphate monobasic dihydrate, dithiothreitol (DTT), and meta-phosphoric acid (MPA) were obtained from Sigma-Aldrich (St. Louis, MO, USA), see Supplementary Data.

### 2.2. Methods

#### 2.2.1. Emulgel Preparation

The various methods of emulgel formulation were reported [9]. The formulation of emulgel for buccal administration in the research work by Perioli and coworkers was based on polymer dispersion in water, after which the polymeric aqueous dispersion was neutralized and the oil phase was added [26]. Another method, reported by Mohamed and coworkers, includes the formation of an emulsion (o/w or w/o), followed by the addition of a gelling agent to form emulgel [27]. Based on the process of the optimization of chlorphenesin in emulgel by Mohamed and coworkers, the preparation of emulgels in the present research work included separate emulsion and gel preparation. The emulsion was prepared following a standard protocol consisting of separate heating of the oil and water phase, and their consequent mixing using an RW 16 basic propeller rotary laboratory stirrer (IKA Werke) until they cooled to room temperature. In order to prepare the emulgel, the gel phase intended for stirring with the emulsion had to be prepared in advance. For that purpose, hydroxyethyl cellulose (HEC) was dispersed in water earlier and left overnight. Finally, a solution of active substances (ascorbyl palmitate and magnesium ascorbyl phosphate) in isopropyl myristate and propylene glycol was respectively added to the emulgel at a temperature of 40 °C. A placebo emulgel (PE) was prepared in the same manner, with the exception that the active substances were not added (Table 1).

#### 2.2.2. Physico-Chemical Testing of Emulgels

The formulated emulgels containing L-ascorbic acid derivatives were subjected to physico-chemical testing. They were analyzed organoleptically (color, thickness, look, feel) and physically (creaming and phase separation). The pH and electrical conductivity values of freshly prepared emugels and of emulgels after a centrifuge assay (at 25 °C and at 3000 rpm for 15 min) were determined. The measurement of the pH value of the investigated emulsions was carried out by the potentiometric method by directly immersing the electrode of a pH meter (pH 211 Microprocessor pH Meter, Hanna Instruments, Woonsocket, Rhode Island, USA) in the prepared samples at a temperature of 22 ± 2 °C. These tests were also carried out after emulgel stability tests (a centrifugation, real-time and accelerated aging test) (Ph Jug. IV, 1984). The electrical conductivity of the investigated emulgels was measured by conductometry, i.e., by direct immersion of the conductometer electrode (CDM 230, Radiometer, Denmark) into the samples and automatically reading the specific conductivity on a digital indicator at a temperature of 22 ± 2 °C. A real-time aging stability test was conducted after 6 months of storage of the emugels at room temperature. The accelerated aging test was performed as follows: samples were stored at room temperature (21 ± 2 °C) for 24 h, then at a temperature of 5 ± 2 °C, and then at a temperature of 45 ± 2 °C. Three such cycles were performed. After the last cycle, the samples were analyzed organoleptically and the pH and conductivity were measured.

#### 2.2.3. Texture Profile Analysis (TPA)

The texture profile analysis (TPA) was performed on a CT3 Texture Analyzer (Brookfield, AMETEK Inc., Middleborough, MA, USA). The following experimental conditions were set: the test speed was set at 2 mm/s, the target value was 2 mm, and the trigger load was 10 g. Each sample was placed in a sample cup, with 75% of each cup filled while avoiding air entrapment. For the analysis cone probe, TA-STF was used, and hardness cycle 1, hardness cycle 2, and cohesiveness and adhesiveness were measured. A TPA was performed in triplicate and the results were presented as the mean values ± standard deviations.

#### 2.2.4. Release Study of L-Ascorbic Acid Derivatives from the Emulgels

The Franz cells (*n* = 6; Gauer Glas, D-Püttlingen, Germany) were used for performing the in vitro release study of L-ascorbic acid derivatives from the formulated emulgels [28]. The chamber volume was 12 mL, while the effective diffusion area was 2.01 cm^2^. Each receiver compartment was covered with the polycarbonate membranes (Nuclepore™, Whatman, Maidstone, United Kingdom; pore diameter: 0.1 μm). The donor chamber was covered with silicone film (Parafilm TM, Neenah, WI, USA) the purpose of which was to inhibit evaporation and preserve the formulation during the sampling. The next step involved joining the donor and the acceptor chamber. Exactly 1 g of each investigated emulgel was placed in the donor chambers. The study lasted for 6 h and the temperature of 32 °C was maintained in the water bath where the cells were placed. At six time points (0.5 h, 1 h, 2 h, 3 h, 4 h and 6 h), the aliquots of 600 μL of the acceptor phase were withdrawn and then replaced with the same volume of phosphate buffered saline. In order to determine the content of magnesium ascorbyl phosphate and ascorbyl palmitate, the LC-MS/MS technique was used.

Magnesium ascorbyl phosphate and ascorbyl palmitate content in the prepared sample solutions were measured according to the method of [29,30]. A Thermo Scientific (Dionex UltiMate 3000) HPLC system containing degasser units, autosamplers, a column compartment binary pump, a glassy carbon working electrode, and an electrochemical detector were used to obtain the data. The Hibar 125-4 LiCrospher100 RP-18 (5 µm) HPLC column (Merck Millipore, Darmstadt, Germany) was used as the stationary phase, while a phosphate buffer (pH = 3) was used as the mobile phase. The conditions set were: a run time of 10 min, an injection volume of 20 µL, a constant flow rate of 800 µL/min, the applied potential for electrochemical measurements +600 mV, and a separation temperature of 25 °C. For instrument control and collection of the data, a Chromeleon7 Chromatography Data System (Thermo Scientific) was utilized. A standard stock solution of L-ascorbic acid (100 mg/L) was prepared in 10% MPA and kept at −20 °C. The calibration standard solutions were prepared by dilution of the stock standard solution in 10% MPA. The quality control mixture used for monitoring instrument performance was prepared by diluting the standard to concentrations of 5 mg/L. The method’s linearity was determined by using ordinary least-square regression. The calibration curve’s concentration ranged from 0.5 mg/L to 50 mg/L, based on its response from the electrochemical detector, while the correlation coefficient was 0.9996. For the calculation of limits of detection (LOD) and the limits of quantification (LOQ), the following equations were used:

LOD = (3.3 × SD)/a(1)

LOQ = (10 × SD)/a(2)

where SD is the standard deviation of the response (standard error value for the coefficient a) and a is the slope value obtained from the linear regression. The obtained limit of detection was 0.26 mg/L, whereas the limit of quantification was 0.86. The recovery of determination was in the range of 92–108%.

#### 2.2.5. Electrical Capacitance (EC), Trans-Epidermal Water Loss (TEWL) and pH Measurements

The effects of examined emulgels on the skin were estimated over a 30 day long-term in vivo study by measuring the skin pH, and the electrical capacitance of the *stratum corneum* (EC) and trans-epidermal water loss (TEWL) using a Multi Probe Adapter MPA^®^9 (Courage & Khazaka Electronic GmbH, Köln, Germany) with different probes attached (Corneometer^®^CM 825, Tewameter^®^TM 300 and skin-pH-Meter PH 905). The study included 20 healthy volunteers (15 women and 5 men) with a mean age of 26.45 ± 6.88 years. None of the participants had a history of skin diseases or had used systemic or topical therapy two weeks before the study began. The participants were instructed to apply the tested emulgels on the specific test-sites twice a day (morning and evening) for 28 days, and not to use any other product on the inside of the forearm during the study. Half of them applied emulgels containing hydrosoluble L-ascorbic acid, and the other half applied emulgels containing lipophilic L-ascorbic acid, at different concentrations, in the same manner. The specific area was intended for placebo emulgel (PE) application, and there was also an area defined as a non-treated control (NC). The measurements were taken after a 30-min rest period with uncovered forearms, at a room temperature of 21 ± 2 °C and a relative humidity of 45 ± 3%. The measurements were performed at several time points, namely after 7, 14, and 28 days of sample utilization in the morning, before any other preparations were applied. The final measurement was conducted 2 days after the application of the samples was discontinued. All participants were informed about the study protocol and signed the written informed consent form before they participated in the study.

#### 2.2.6. Melanin Index (MI) Measurements

In vivo measurements of melanin index (MI) were performed by using a Multi probe adapter 9 device, (Courage & Khazaka Electronic GmbH, Germany) and using the appropriate probe, a Mexameter^®^ MX 16.

In the study, 14 healthy volunteers (mean age 24.93 ± 3.03 years), of both genders, with moderately dry skin (the condition for participation in the experiment is a subjective assessment), with no history or clinical signs of dermatological diseases participated. The survey was conducted in November. It was emphasized to subjects that they should not use any skin care products either during the study or for one week before the start of the study. Furthermore, they should not use any skin care products in the examined area. The test of the ability of the investigated emulgels to lighten the hyperpigmentation on the skin was carried out in accordance with the guides and earlier scientific publications [31,32,33,34].

After the initial measurement (basal values), a commercial emulsion lotion containing 3% of dihydroxyacetone (DHA) in the amount of 1 mL was applied to the inside of the volunteers’ forearms. The treated areas were left uncovered until the skin had completely absorbed the DHA. The measurement was performed again, 24 h later. The volunteers started to apply differently labeled samples, in the amount of a pea, daily (morning and evening) for 7 days to precisely determine the places on the volar side of the forearms. Before the beginning of the measurements, they received information about the place on the skin to which each of the samples should be applied, in addition to the amount and method of application. The measurements were performed 3 and 7 days after the application of the samples, in the morning, and before the morning application.

#### 2.2.7. Examination of Sensory Properties

The sensory evaluation was carried out in conditions that imply a properly illuminated laboratory, a room temperature of 21 ± 2 °C, and a relative humidity of 45 ± 3%. Certain sensory characteristics of the investigated emulgel formulations were assessed by a questionnaire used in our previous studies (Table 2) [1,35]. In the study, 20 participants evaluated the characteristics of the samples before, during, and after application by selecting descriptive terms from a provided list of sensory attributes. The results were then presented using an ordinal scale and recalculated so that the strongest attribute has a value of 10.

#### 2.2.8. Ethical Standards

The entire study was conducted in accordance with the Helsinki Declaration, and the guidelines and published recommendations while permission was provided by the Ethics Committee of the Medical Faculty in Niš (Serbia), protocol code 12-10650/2-7 from the 3 October 2022.

#### 2.2.9. Statistical Analysis

The obtained results of in vivo measurements and the textural analysis for each parameter were presented as mean ± standard error. The obtained values of the samples with active substances were compared to basal ones, to the placebo samples, and to the non-treated control. In addition, any changes in the measured values during the study were analyzed. Active emulgels were compared to each other in order to examine the influence of the emulgel as a carrier system on the effects of the L-ascorbic acid derivatives of different polarities. A statistical analysis was performed using a One-way analysis of variance ANOVA with a post-hoc Tukey’s test by using IBM SPSS Statistics 20 (IBM Corporation, Tokyo, Japan). Differences at *p* < 0.05 were considered statistically significant.

## 3. Results and Discussion

When developing a topical formulation, it is important to select a carrier system that is compatible with the active ingredients and is appropriate for the intended use of the product [36]. Some products placed on the market containing unstable free forms of L-ascorbic acid require great investments in specific methods of packaging and containers in order to prolong formulation stability. Therefore, the future lies in the development of stable and safer new delivery systems, such as microemulsions, emulgels, microcapsules, nanospheres, and liposomes. Additionally, the usage of L-ascorbic acid derivatives such as magnesium ascorbyl phosphate and sodium ascorbyl phosphate (water-soluble derivatives), and ascorbyl-6-palmitate and tetra-isopalmitoyl ascorbic acid (liposoluble derivatives) are increasingly replacing the use of the free L-ascorbic acid form [37]. Studies comparing the stability of the L-ascorbic acid derivatives have demonstrated that of all forms incorporated in solutions or emulsions, magnesium ascorbyl phosphate was the most stable one, followed by ascorbyl-palmitate, whereas the L-ascorbic acid was the least stable [13]. Ascorbyl palmitate, an amphiphilic ascorbic acid derivative, has better stability and ability to penetrate the skin than L- ascorbic acid [38].

The hydroxyethyl cellulose- (HEC) based emulgels showed good drug release profiles and rheological characteristics [9] and optimal textural properties compared to other cellulose polymers [39]. Considering the advantageous characteristics of HEC emulgel application, in the presented work this type of emulgel was selected as a carrier system for the dermal delivery of L-ascorbic acid derivatives, ascorbyl palmitate and magnesium ascorbyl phosphate. A literature survey revealed that there were no studies comparing the L-ascorbic acid water-soluble and liposoluble derivatives when vehiculated in the form of an emulgel.

The expected organoleptic stability of creams was achieved in terms of color, thickness, and appearance. No phase separation after centrifugation was found in any of the investigated samples. The values of electrical conductivity and pH, before and after the centrifugal test, as well as after accelerated and long-term stability test were determined, and are reported in Table 3. The table pH value, as well as the preserved electrical conductivity value of the tested emulgels, were the indicators of satisfactory preliminary physical and chemical stability of the investigated formulations, given that they were at almost the same level before and after the centrifugation test [40,41]. During the long-term stability study (real-time aging), the only noticeable change occurred in ASP1 and ASP2 (emulsions containing the lipophilic form of L-ascorbic acid), which changed color from white to light yellow after four months of storage at room temperature.

During the formulation and development of topical preparations, a very important aspect is their textural characterization, which is largely responsible for the acceptability of that preparation by the user/patient [42]. The sensory analysis is a subjective analysis involving volunteers who, after applying the product to the skin, give their impression of the product’s properties. On the other hand, textural analysis is an objective analysis, in which the device-texture analyzer, with the probe that simulates a human finger touching the product, gives a force-time graph based on which the values of textural parameters can be calculated, such as cohesiveness, resilience, and adhesiveness [43,44]. Texture Profile Analysis (TPA) is the most popular and widely used method of texture analysis of semi-solid products today, and is used to relate the mechanical characteristics of samples to their sensory characteristics [45]. The choice of the preparation is greatly influenced by its properties during application and removal from the packaging [42], which was previously confirmed by comparing the results of the textural and sensory analysis [35]. In our earlier work, the influence of the choice of thickeners on textural analyses of oil-in-water emulsions was examined [39]. The results showed that hydroxyethyl cellulose had the most desirable properties of the analyzed thickeners. Therefore, it was used in the preparation of emulgels. The results of the texture analysis of the emulgels in this study are presented in Table 4.

Adhesiveness is an indicator of the stickiness of the product to other surfaces [46]. The results showed that the hydro-lipophilic properties of L-ascorbic acid as an active substance generally did not affect the adhesiveness of the tested emulgels. The only statistically significant difference was observed between PE and ASP2 (*p* < 0.05).

A similar finding was observed in the case of cohesiveness, where none of the tested samples were significantly different from the others (*p* < 0.05). Neither the addition of L-ascorbic acid as an active substance nor the difference in its lipophilicity influenced the cohesiveness. Cohesiveness signalizes the strength of the internal bonds of the products. Mathematically, it is calculated as the ratio of work performed during two cycles and therefore has no units [47].

Hardness, as a force required for deformation [43], was measured during both cycles (hardness cycle 1 and hardness cycle 2). Higher values of hardness are associated with emulgels that are more difficult to apply on the skin since the hardness describes the force required to rub a product between fingers [46]. The results have shown that emulgels MAP1 and MAP2 are more difficult to spread than others (*p* < 0.05). Since there were no statistically significant differences between the Hardness Cycle 1 and Hardness Cycle 2 values, it can be concluded that the weakening of the emulgel structure was not observed, and that the deformation did not disrupt the structure of the samples, which is an indicator of the stability of the emulgels.

**Figure 1:**
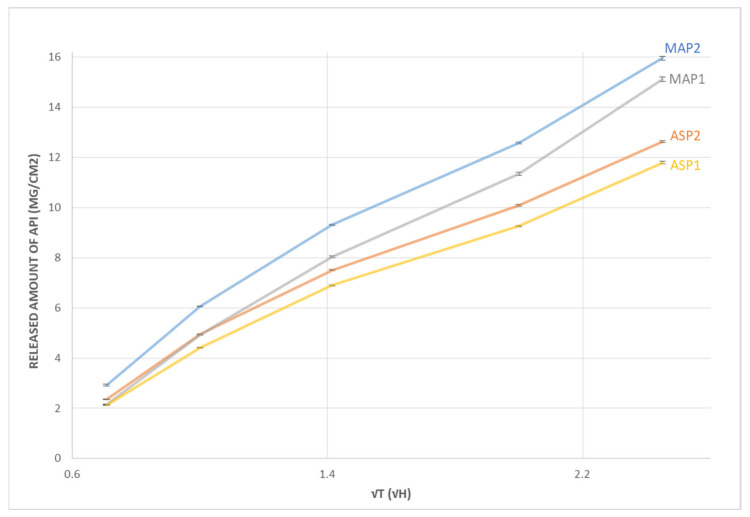
Release profiles of L-ascorbic acid derivatives from investigated emulgels (ASP1, MAP1, ASP2 and MAP2).

The release profiles of the active substance (L-ascorbic acid derivatives) from the investigated emulgels are shown in Figure 1. The best release was observed in emulgel MAP2, while the lowest release was associated with ASP1. In general, emulgels with a hydrophilic active substance showed a better release profile than those with a lipophilic form of L-ascorbic acid, indicating that the hydrophilicity of the active substance affects the release profile. All samples followed Higuchi kinetics, which is typical for semi-solid preparations [48].

Although the results of the textural analysis showed that emulgels with a hydrophilic active substance (MAP1 and MAP2) had higher hardness values, they revealed the better release of the active substance, which is not typical, while it is generally accepted that the active substance is released more slowly from more viscous formulations [49]. However, there are other examples in the literature where this was not the case, emphasizing the fact that the release of active substances from semi-solid formulations is a rather complex process [50].

Franz diffusion cell release experiments are commonly used to uncover the mechanism of active ingredient release and to determine the potential of a particular carrier system for trans-membrane delivery. Jurkovič et al. investigated the release of ascorbyl palmitate from four different microemulsions (w/o, o/w with or without a thickening agent) using a cellulose acetate membrane. The authors found that there was a statistically significant difference between the liberation speed of the lipophilic L-ascorbic acid derivative from different systems since release from o/w microemulsions was slower [51]. Gosenca et al. [52] investigated the suitability of various colloidal systems (microemulsions and liquid crystals) as carriers for ascorbyl palmitate delivery into the skin. They found that the liquid crystal was the most adequate system when taking into account both the ascorbyl palmitate solubilization capacity and the permeation effect. The best results regarding the skin permeation were observed for liquid crystal-loaded with 1% ascorbyl palmitate when the release profile was determined in the Franz cell diffusion study. In the study by Ćorović et al. [53], it was demonstrated that there was a significant influence of formulation type on the trans-membrane diffusion of fatty acid ascorbyl esters. Comparing classical o/w emulsion and gel-emulsion with incorporated coconut oil-derived fatty acid ascorbyl esters, trans-membrane delivery of the same molecule was achieved more rapidly from the gel-emulsion, with a higher diffusion rate and approximately 2–3 times higher amounts of delivered active compounds. Previous research has shown that the release rate of L-ascorbyl palmitate from cosmetic preparations is dependent on the formulation used [54]. This was further confirmed in the study by Rahman et al. Namely, the effectiveness of three formulations (gel-like microemulsion, liquid o/w microemulsion, and conventionally thickened o/w microemulsion) as the vehicles for simultaneous topical delivery of L-ascorbic acid and vitamin E was investigated using an in vitro skin permeation test. The release profiles of vitamins at skin temperature from examined types of formulations were also determined and compared. It was demonstrated that the vitamins’ release from gel-like microemulsions was comparable to those from o/w microemulsions. In addition, the much faster and more complete release profiles of vitamins were achieved from the gel-like microemulsion compared to the o/w microemulsion conventionally thickened with carbomer. [55].

Iliopoulos et al. investigated the physicochemical properties of 3-o-ethyl-l-ascorbic acid, another stable L-ascorbic acid derivative that can be used in products intended for dermal application. They also examined the impact of different solvents (either hydrophilic or lipophilic) on its delivery and in vitro skin permeation through porcine skin using a Franz diffusion cell. Through the results of their study, they confirmed that the choice of the solvents in formulation influences the delivery of L-ascorbic acid derivatives to the skin [56].

According to the study by Farahmand et al. [57], the release profile of L-ascorbic acid from multiple emulsions follows zero-order kinetics. In the first four-hour period, about 14% of the molecule was released from o/w/o emulsions. Due to low partitioning and solubility of L-ascorbic acid in the external oily phase of the multiple systems, the external oil phase acts as a barrier for diffusion of a hydrophilic, freely soluble compound, while the migration of L-ascorbic acid from the inner aqueous phase was delayed.

When hydrosoluble derivatives of L-ascorbic acid were used in a topical formulation, Špiclin et al. revealed that the difference in release profiles of sodium ascorbyl phosphate incorporated in microemulsions depended on the type and the presence of thickening agent. The authors showed the continuous release behavior of the sodium ascorbyl phosphate from the w/o microemulsions. Compared to the o/w systems, less ascorbyl phosphate was liberated from both w/o microemulsions with and without thickeners. The presence of a thickening agent and the location of sodium ascorbyl phosphate in the microemulsions significantly influenced its release profiles. For instance, xanthan gum caused the increase of the viscosity of the system, and consequently the decreased amount of released active substance. However, the colloidal silica showed the opposite effect. It was found that obtained experimental data follow Higuchi kinetics, by fitting them to different order kinetic equations, which is in accordance with the results obtained in our study [38].

A topical delivery system of L-ascorbic acid nanoparticles of varying concentrations incorporated into the hydroxypropyl methylcellulose (HPMC) gels exhibited sustained release over 8 h. The drug release was shown to depend on the polymer concentration, with a higher concentration of HPMC leading to a decrease in drug release from the L-ascorbic acid nanoparticle gel [58].

A 30-day in vivo study was conducted on healthy volunteers to assess the impact of the evaluated emulgels on human skin by measuring the following skin parameters with different aims:

- electrical capacitance (EC) was an indicator of the effect of tested emulgels on skin moisture;
- transepidermal water loss (TEWL) was measured to determine whether the tested emulgels influence skin barrier function;
- skin pH to assess the effect of tested emulgels on skin damage;
- melanin index (MI) portrayed the effects of the tested emulgels on skin color and the possibility of the lightening of the skin.

**Figure 2:**
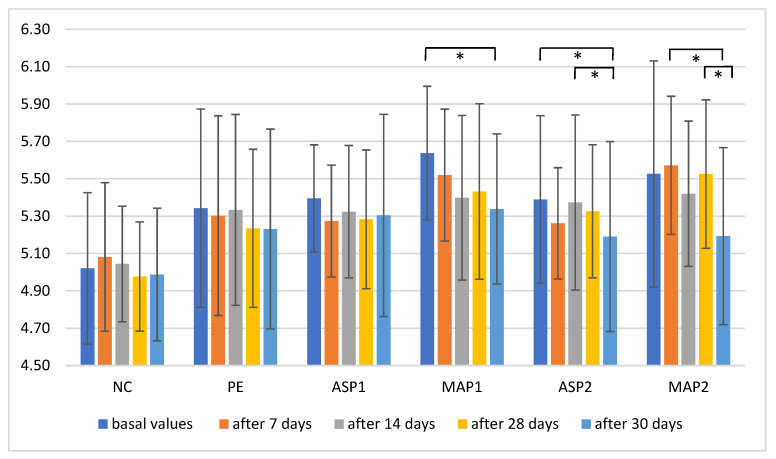
In vivo determined mean values with standard deviation of pH for non-treated control (NC), placebo emulgel (PE), and the investigated emulgels (ASP1, MAP1, ASP2 and MAP2) after 7, 14, and 28 days of application, and 2 days after the cessation of application. Significant differences are marked with * (*p* < 0.05).

The pH is an important parameter regarding the effectiveness and stability of topical preparations. The pH of the skin ranging between 4 to 6.5 and 5.5 represents the average pH of the skin [59]. Our results revealed that during the study, a drop in pH value was observed, which may be a consequence of the acidic properties of L-ascorbic acid derivatives (Figure 2). However, the only statistically significant changes (*p* < 0.05) in pH compared to basal values were noticed after 30 days of application of MAP1 and ASP2.

**Figure 3:**
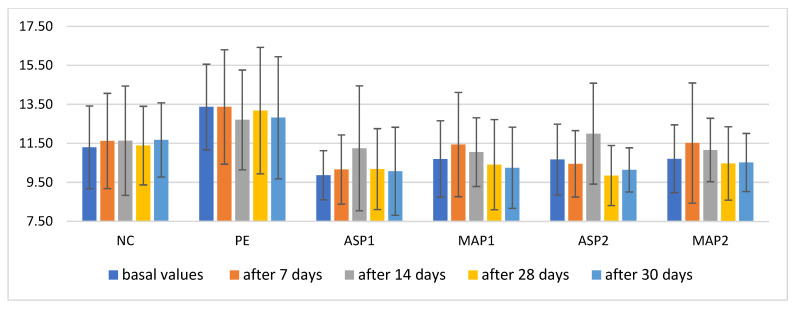
In vivo determined mean values with a standard deviation of trans-epidermal water loss (TEWL) for non-treated control (NC), placebo emulgel (PE) and investigated emulgels (ASP1, MAP1, ASP2 and MAP2) after 7, 14, and 28 days of application, and 2 days after the cessation of application.

TEWL is considered to be an important measure of epidermal barrier function. During the study (Figure 3), there were no statistically significant changes in TEWL after the application of the tested samples, which meant that the integrity of the *stratum corneum* was not interrupted during the usage of the preparations.

The *stratum corneum* moisture content was determined by monitoring the skin capacitance. This non-invasive method involves measuring electrical capacitance, based solely on the water content in the skin. Changes in capacitance are translated into digital values that reflect the skin’s moisture level. [60].

**Figure 4:**
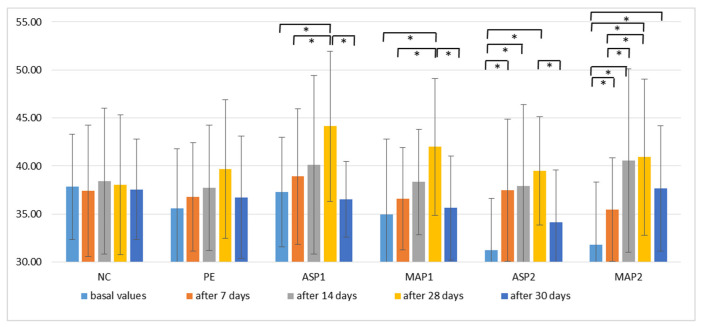
In vivo determined mean values with a standard deviation of electrical capacitance (EC) for non-treated control (NC), placebo emulgel (PE) and investigated emulgels (ASP1, MAP1, ASP2 and MAP2) after 7, 14, and 28 days of application, and 2 days after the cessation of application. Significant differences are marked with * (*p* < 0.05).

During the application of active emulgels, an increase in the degree of hydration of the skin was observed (Figure 4). Compared to the basal values, all active emulgels showed a statistically significant increase in hydration after 28 days (*p* < 0.05). There were no statistically significant changes in EC parameters during the application of the placebo emulgel as well as in the untreated area. Two days after stopping the use of active emulgels, a drop in hydration was observed between the 28th and 30th days, which were statistically significant for all emulgels except for MAP2. Bearing in mind that the highest quantity of the drug was released from the sample MAP2, it may be speculated that such a finding was connected to the highest increase in hydration of the *stratum corneum* observed after treatment with this sample (Figure 1). However, further in vivo investigations of skin penetration are needed for the confirmation of such assumptions. Namely, although there was no statistically significant difference in the increase of EC level between the sites treated with different active emulgels, a trend of increased moisture of the skin can be observed in the MAP2-treated site compared to the others, including non-treated control and placebo. Similarly, it was previously shown that formulations with magnesium ascorbyl phosphate enhanced the *stratum corneum* moisture content after a 4-week period of daily applications when compared to the baseline values. Significant effects have been observed in improving the condition of the skin by increasing the hydration of the skin in the deeper layers of cells [37].

**Figure 5:**
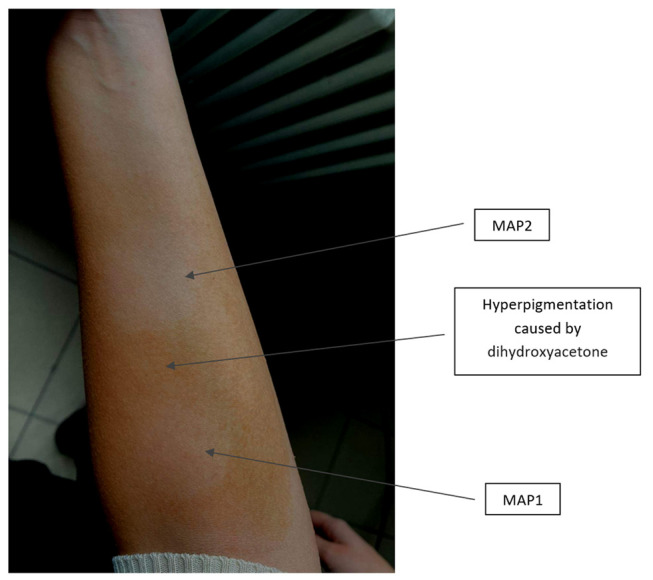
Hyperpigmentation caused by dihydroxyacetone and lightening of the skin caused by the application of MAP1 and MAP2.

**Figure 6:**
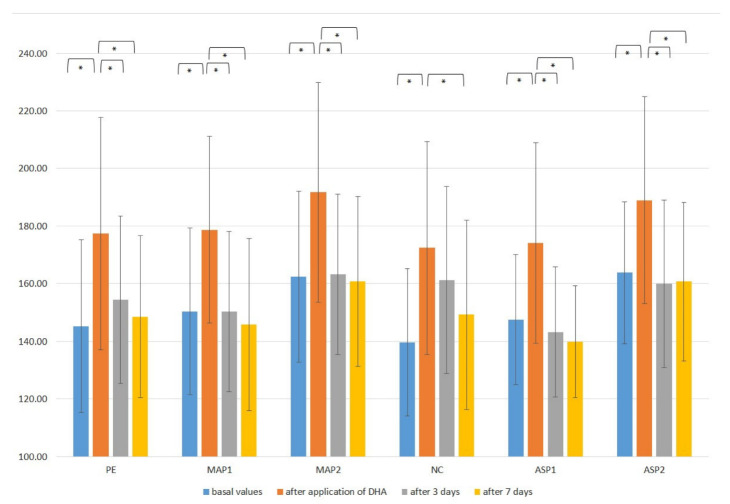
In vivo determined mean values with a standard deviation of melanin index (MI) for non-treated control (NC), placebo emulgel (PE) and investigated emulgels (ASP1, MAP1, ASP2 and MAP2) after 3 and 7 days of application. Significant differences are marked with * (*p* < 0.05).

The melanin index (MI) was determined in order to evaluate the effects of examined emulgels on skin color and its lightening. The results showed a tendency for the tested emulgels to lighten the skin (Figure 5). The MI parameter significantly decreased (*p* < 0.05) at all test sites (ASP1, MAP1, ASP2 and MAP2) after 7 days of the study. (Figure 6).

Combined treatment with active multiple emulsion formulations containing ascorbyl palmitate and sodium ascorbyl phosphate produced synergistic effects on the skin melanin and erythema [59,61]. Thus, this kind of formulation could be explored further for the treatment of pigmentation disorders and dermatitis. Topically applied magnesium-L-ascorbyl-2-phosphate cream on human skin caused a significant lightening of melasma and lentigines, exhibiting the beneficial effects in hyperpigmentation lightening [62].

Over the last few years, there has been a constant development in the field of sensory analysis. It has been extensively used to describe and quantify the texture characteristics of cosmetic products. The sensory analysis represents a helpful tool for the research and development of companies, enabling the formulation of cosmetic products with good consumer acceptance. Besides that, the data obtained by sensory characterization contributes to the formulation of topical products characterized by predefined sensory features or in the process of reformulation [63].

**Figure 7:**
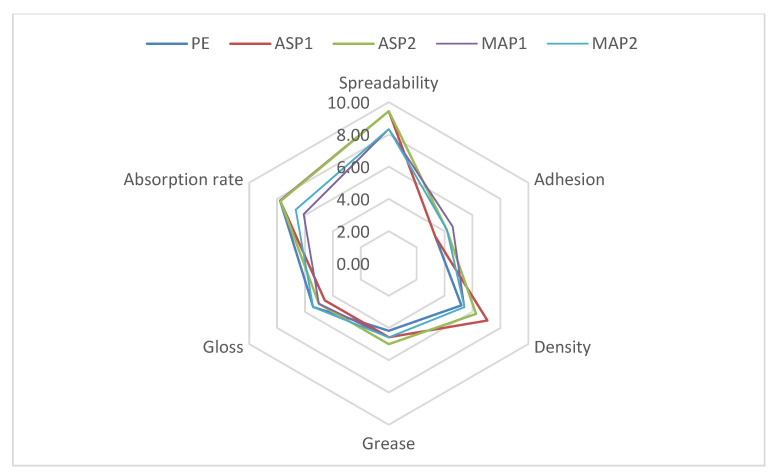
The results of the sensory analysis of the investigated samples during application.

**Figure 8:**
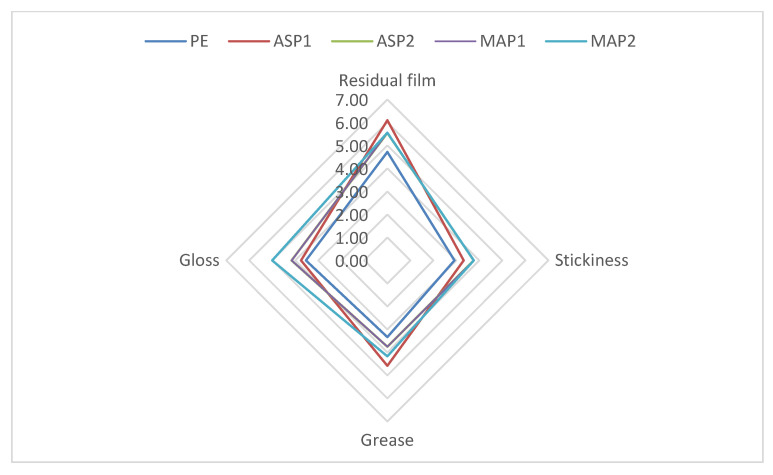
The results of sensory analysis of the investigated samples after application.

The participants were asked to complete a questionnaire on product sensory characteristics prior to, during, and following application on the skin. Most of the volunteers reported that the tested emulgels had a semi-solid consistency, were non-greasy, and were easy to spread (Table 5). All participants concurred that emulgels had a mild stickiness, firmness, and consistency (Figure 7). After application, the results showed that there was a moderate film left with a slight sheen on the skin. However, there was no greasy nor sticky feeling on the skin. All respondents agreed that emulgels were moderately absorbed, except the emulgel containing ascorbyl palmitate at a concentration of 1% *w/w*, which was labeled as one that was fast absorbed (Figure 8).

It can be concluded that the hydro-lipophilic properties of L-ascorbic acid derivatives did not affect the sensory characteristics of the investigated emulgels.

## 4. Conclusions

Despite its various proven biological activities, L-ascorbic acid represents a challenge when it comes to its incorporation into topical (dermocosmetic) formulations. The emulgel containing hydrophilic L-ascorbic acid derivative showed the best properties in terms of the release profile of the active substance and the effect on the biophysical parameters of the skin. According to the results of our study, the most prolonged hydration of the skin was observed in the place treated with the sample MAP2 containing 2% of hydrophilic magnesium ascorbyl palmitate, even after the cessation of the sample application. However, different concentrations and types of L-ascorbic acid derivatives incorporated in the emulgel did not influence the texture and sensory characteristics of the preparations. Nevertheless, the difference in hydrophilic/lipophilic properties of L-ascorbic acid derivatives did not affect the sensory and textural characteristics of the investigated emulgels. Based on the results of our study, formulated emulgels may be considered as stable delivery systems for L-ascorbic acid derivatives (in particular magnesium ascorbyl phosphate), with the appropriate sensory characteristics and the potential for skin hydration and hypopigmentation.

The subject of our future work would be based on establishing the protocol for the in vitro permeation studies on porcine ear skin and tape-stripping as an in vivo study in order to complete the release and permeation profile of active substances from formulated emulgels. Further research should be undertaken in order to investigate the potential therapeutic relevance of the developed emulgels.

## Figures and Tables

**Table 1:** Qualitative and quantitative compositions (%, (*w*/*w*)) of emulgel samples.

Ingredients (INCI Name)	Function in the Formulation	Content [% _m/m_]				
		ASP1	ASP2	MAP1	MAP2	PE
**Oil phase**						
Caprylic/capric triglycerides	Emollient	11.00	11.00	11.00	11.00	11.00
Isopropyl myristate	Emollient	7.50	7.50	7.50	7.50	7.50
Olive oil	Emollient	3.00	3.00	3.00	3.00	3.00
Cetearyl alcohol (and) Coco-glucoside	o/w emulsifier	7.00	7.00	7.00	7.00	7.00
Myristyl alcohol (and) Myristyl glucoside	o/w emulsifier	1.50	1.50	1.50	1.50	1.50
**Active substance**						
Ascorbyl palmitate		1.00	2.00	-	-	-
Magnesium ascorbyl phosphate		-	-	1.00	2.00	-
**Water phase**						
Hydroxyethyl cellulose (HEC)	Thickener/Gelling agent	1.00	1.00	1.00	1.00	1.00
Propylene glycol	Humectant	10.00	10.00	10.00	10.00	10.00
Phenoxyethanol (and) Ethylhexylglycerin	Preservative	1.00	1.00	1.00	1.00	1.00
Aqua (Water)	Water phase	q.s. 100.00	q.s. 100.00	q.s. 100.00	q.s. 100.00	q.s. 100.00

ASP1, ASP2, MAP1 and MAP 2 indicate emulgels containing 1% and 2% *w/w* of ascorbyl palmitate and magnesium ascorbyl phosphate, respectively; PE: Placebo emulgel.

**Table 2:** Sensory evaluation questionnaire.

Prior to Application							
**Consistency**				**Gloss Level**			
liquid				matte		gloss	
semi-solid				pearl gloss		very glossy	
				slightly glossy			
**In the Course of Application**							
**Spreadability**	**Adhesion**		**Density**	**Grease**	**Gloss**		**Absorption rate**
very difficult to spread	not sticky		rare	not greasy	not shiny		slow
difficult to spread	slightly sticky		slightly dense	slightly greasy	slightly shiny		moderate
easy to spread	sticky		dense	greasy	shiny		fast
	very sticky		very dense	very greasy	very shiny		
**Following Application**							
**Residual film**		**Stickiness**		**Grease**		**Gloss**	
no film		not sticky		not greasy		not shiny	
moderate film		slightly sticky		slightly greasy		slightly shiny	
expressive film		sticky		greasy		shiny	
		very sticky		very greasy		very shiny	

**Table 3:** pH and electrical conductivity (μS/cm) values of emulgel samples before and after a centrifuge assay, as well as after an accelerated and long-term stability test.

pH				
	Before Centrifuge Assay	After Centrifuge Assay	After Accelerated Stability Test	After Long-Term Stability Test
**MAP 1**	7.16	7.17	*7.14*	*7.16*
**MAP 2**	7.17	7.20	*7.15*	*7.16*
**ASP 1**	4.21	4.23	*4.24*	*4.20*
**ASP 2**	3.99	3.94	*3.98*	*4.00*
**Electrical conductivity (μS/cm)**				
	**Before** **centrifuge assay**	**After** **centrifuge assay**	***After*** ***accelerated*** ***stability test***	***After*** ***long-term*** ***stability test***
**MAP 1**	−4.4	−5.5	*−4.9*	*−4.4*
**MAP 2**	−5.4	−7.2	*−6.8*	*−5.9*
**ASP 1**	166.5	164.9	*165.9*	*165.4*
**ASP 2**	178.7	182.1	*184.2*	*179.0*

**Table 4:** The results of the texture analysis of tested emulgels.

	Adhesiveness (mJ)	Cohesiveness	Hardness Cycle 1 (g)	Hardness Cycle 2 (g)
PE	0.60 ± 0.10 ^a^ *	0.80 ± 0.19 ^a^	23.33 ± 1.15 ^b^	21.67 ± 1.53 ^b^
MAP1	0.45 ± 0.07 ^ab^	0.89 ± 0.02 ^a^	36.00 ± 5.66 ^a^	35.50 ± 6.36 ^a^
MAP2	0.50 ± 0.00 ^ab^	0.86 ± 0.05 ^a^	33.67 ± 4.16 ^a^	31.67 ± 4.04 ^a^
ASP1	0.47 ± 0.06 ^ab^	0.87 ± 0.14 ^a^	21.67 ± 1.53 ^b^	20.67 ± 1.53 ^b^
ASP2	0.43 ± 0.06 ^b^	1.07 ± 0.11 ^a^	21.67 ± 0.58 ^b^	21.00 ± 0.00 ^b^

* Means followed by a common letter in the column are not significantly different at the 95% level of significance (*p* < 0.05).

**Table 5:** The results of sensory analysis of the investigated samples before application converted to 1–10 scale.

Before Application					
	PE	ASP1	ASP2	MAP1	MAP2
Consistency	8.32	9.17	9.17	6.64	10.00
Gloss level	5.83	5.67	6.33	5.66	5.33

## Data Availability

The data presented in this study are contained in the article.

## Supplementary Materials

The following supporting information can be downloaded at: https://www.mdpi.com/article/10.3390/pharmaceutics15030813/s1, Supplementary Data [9,39,64].
