# Supplementary material for: Derivatives of L-Ascorbic Acid in Emulgel: Development and Comprehensive Evaluation of the Topical Delivery System
Source: Pharmaceutics. 2023 Mar 2;15(3):813. doi: 10.3390/pharmaceutics15030813 (PMC10056080; doi:10.3390/pharmaceutics15030813)
Supplement: Supplementary file 1 [file pharmaceutics-15-00813-s001.zip › pharmaceutics-2121956-supplementary.pdf]

Derivatives of L-Ascorbic Acid in Emulgel: Development and Comprehensive Evaluation of the Topical Delivery System

Aleksandra Stolić Jovanović [1], Milica Martinović [2], Ana Žugić [3], Ivana Nešić [2], Tomislav Tosti [4], Stevan Blagojević [5] and Vanja M. Tadić [3,*]

[1] "Filly Farm" Pharmacy, Miloša Velikog bb, 11320 Velika Plana, Serbia
[2] Department of Pharmacy, Faculty of Medicine, University of Nis, Boulevard Dr. Zorana Djindjića 81, 18000 Nis, Serbia
[3] Department for Pharmaceutical Research and Development, Institute for Medicinal Plant Research "Dr. Josif Pančić", Tadeuša Koscuška 1, 11000 Belgrade, Serbia
[4] Faculty of Chemistry, University of Belgrade, Studentski trg 12-16, 11158 Belgrade, Serbia
[5] The Institute of General and Physical Chemistry, Studentski trg 12/V, 11158 Beograd, Serbia
* Correspondence: vtadic@mocbilja.rs

Supplementary Data

HEC was used to formulate the gel phase of the emulgel and also serves as a thickening agent. HEC based emulgel is a prototype of the formulation already available. In our previous work (Stolić-Jovanović, A.; Martinović, M.; Nešić, I. The Influence of Selected Thickeners on the Textural Properties of Oil-In-Water Emulsions. Acta Fac. Medicae Naissensis 2022, 39(1), 57-65.), the hydroxyethyl cellulose (HEC) based emulgels showed optimal textural properties compared to other cellulose polymers. Also, they have a good drug release profiles and rheological characteristics (Ajazuddin, A.A.; Khichariya, A.; Gupta, C.; Patel, J.R.; Kumar, G.T.; Krishna, T.D. Recent expansions in an emergent novel drug delivery technology: Emulgel. J Control Release 2013, 171(2), 122–132.)

Caprylic/capric triglycerides were selected as the major part of the oil phase due to their emollient characteristics.

Isopropyl myristate is widely used mineral oil both as the vehicle for the drug and for its occlusive and sensory characteristics, usually in quantity of 7-7,5% (Yadav, S. K., Mishra, M. K., Tiwari, A., & Shukla, A. (2016). Emulgel: a new approach for enhanced topical drug delivery. Int J Curr Pharm Res, 9(1), 15-19.).

Oleic acid is widely used in pharamaceuticals, which increases percutaneous drug absorption. It is the most abundant unsaturated long-chain fatty acid and an important ingredient of olive oil, hence we decided to use it for emulgel formulation.
